# Box–Behnken Response Surface Design of Polysaccharide Extraction from *Rhododendron arboreum* and the Evaluation of Its Antioxidant Potential

**DOI:** 10.3390/molecules25173835

**Published:** 2020-08-24

**Authors:** Ajaz Ahmad, Muneeb U. Rehman, Adil Farooq Wali, Hamed A. El-Serehy, Fahad A. Al-Misned, Saleh N. Maodaa, Hossam M. Aljawdah, Tahir Maqbool Mir, Parvaiz Ahmad

**Affiliations:** 1 Department of Clinical Pharmacy, College of Pharmacy, King Saud University, Riyadh 11451, Saudi Arabia; mrehman1@ksu.edu.sa; 2 Department of Pharmaceutical Chemistry, RAK College of Pharmaceutical Sciences, RAK Medical and Health Science University, Ras Al Khaimah 11172, UAE; farooq@rakmhsu.ac.ae; 3 Department of Zoology, College of Science, King Saud University, Riyadh l1451, Saudi Arabia; helserehy@ksu.edu.sa (H.A.E.-S.); fahadaalmisned@gmail.com (F.A.A.-M.); snmaodaa@ksu.edu.sa (S.N.M.); hmaljadah@ksu.edu.sa (H.M.A.); 4 National Center for Natural Products Research, Research Institute of Pharmaceutical Sciences, School of Pharmacy, University of Mississippi, Oxford, MS 38677, USA; tmmir@olemiss.edu; 5 Department of Botany and Microbiology, College of Science, King Saud University, Riyadh 11451, Saudi Arabia

**Keywords:** *Rhododendron arboreum*, polysaccharides, Box–Behnken design, antioxidant activity

## Abstract

In the present investigation, the ultrasound-assisted extraction (UAE) conditions and optimization of Rhododendron arboreum polysaccharide (RAP) yield were studied by a Box–Behnken response surface design and the evaluation of its antioxidant potential. Three parameters that affect the productivity of UAE, such as extraction temperature (50–90 °C), extraction time (10–30 min), and solid–liquid ratio (1–2 g/mL), were examined to optimize the yield of the polysaccharide percentage. The chromatographic analysis revealed that the composition of monosaccharides was found to be glucose, galactose, mannose, arabinose, and fucose. The data were fitted to polynomial response models, applying multiple regression analysis with a high coefficient of determination value (R^2^ = 0.999). The data exhibited that the extraction parameters have significant effects on the extraction yield of polysaccharide percentage. Derringer’s desirability prediction tool was attained under the optimal extraction conditions (extraction temperature 66.75 °C, extraction time 19.72 min, and liquid–solid ratio 1.66 mL/g) with a desirability value of 1 yielded the highest polysaccharide percentage (11.56%), which was confirmed through validation experiments. An average of 11.09 ± 1.65% of polysaccharide yield was obtained in optimized extraction conditions with a 95.43% validity. The in vitro antioxidant effect of polysaccharides of R. arboreum was studied. The results showed that the RAP extract exhibited a strong potential against free radical damage.

## 1. Introduction

*Rhododendron arboreum* (RA) is a small tree or evergreen shrub of the family Ericaceous. The plant was originally discovered in north India, especially in the Himalayan regions from Kashmir to Bhutan and in the hills of Manipur and Assam at higher altitudes [1]. A literature survey reveals that *R. arboreum* has wide pharmaceutical uses. The plant has shown antioxidant, hepatoprotective, anti-inflammatory, antidiabetic, and anti-diarrheal uses due to the presence of various phytoconstituents such as flavonoids, tannins, and saponins [2,3,4,5]. Fresh petals are processed for the preparation of sub-acidic jelly and sharbat, a well-known market product. *R. arboreum* young leaves cause intoxication if consumed in large quantities, as well as having medicinal uses, and can be applied on the forehead to ease a headache [6,7]. This plant is not listed for its harmful effects [8]; consequently, the likelihood of it harming humans is tremendously low. *R. arboreum* flowers with a sweet and sour taste are used in the preparation of squash, jams, jellies, and local brewing in hilly areas. It can make a very popular and enjoyable cocktail, drank as a soothing appetizer once a day and can even be used to avoid sickness at high altitudes [6]. The dried leaves of *R. arboreum* have been used in gout and rheumatism in the homeopathic method of medicine. In Ayurveda, the *R. arboreum* has a synthetase-inhibiting function and is oxytocic, estrogenic, and prostaglandin [9]. *R. arboreum* dried flowers are reportedly extremely successful in preventing diarrhea and blood dysentery [3]. Polysaccharides are key elements for living objects and remain widely distributed in animals, plants, and the microbial cell walls. In recent times, plant-derived polysaccharides have received interest for their variety of medicinal properties, such as being antioxidant and antitumor; having immunological, antimicrobial, and anti-hyperlipidemic activity; and having almost no adverse effects on human health [10,11,12].

Ultrasonic-assisted extraction (UAE) is a technique that reduces the handling time and consumption of solvents, simplifying operation and work-up, producing a higher quality of final product. This is due to its low temperatures, which reduce the heat loss caused by high temperatures; it also prevents vaporization during boiling and also prevents the preservation of biological active substances. This is very useful in the extraction of heat labile compounds [13]. It was observed from the published data that UAE was investigated to extract polysaccharides from different plant materials, resulting in a significantly reduced extraction time and improved overall targeted compound extraction yield relative to traditional methods [14,15,16]. Response surface methodology (RSM) is an effective mathematical and statistical technique to investigate and optimize complex processes. The Box–Behnken design (BBD), an RSM tool, has been widely used by investigators for the optimization of experimental trials [17]. The Box–Behnken design is advantageous because it does not contain any points at the extremes of the cubic region created by the two-level factorial level combinations that are prohibitively expensive or impossible to test because of physical constraints in experimentation [18]. The Box-Behnken design (BBD) has been widely used in pharmaceuticals, bioprocessing, food engineering, agrochemicals, and other industries to extract biological active compounds, such as polysaccharides, phenolic compounds, and proteins from various sources, intended for human use [14]. Hereafter, in the present investigation, we demonstrate the effect of the UAE of process variables, such as the extraction temperature, time, and solid–liquid ratio, on the percentage yield of RAP using BBD, and its in vitro anti-oxidant potential.

## 2. Results and Discussion

**Figure 1:**
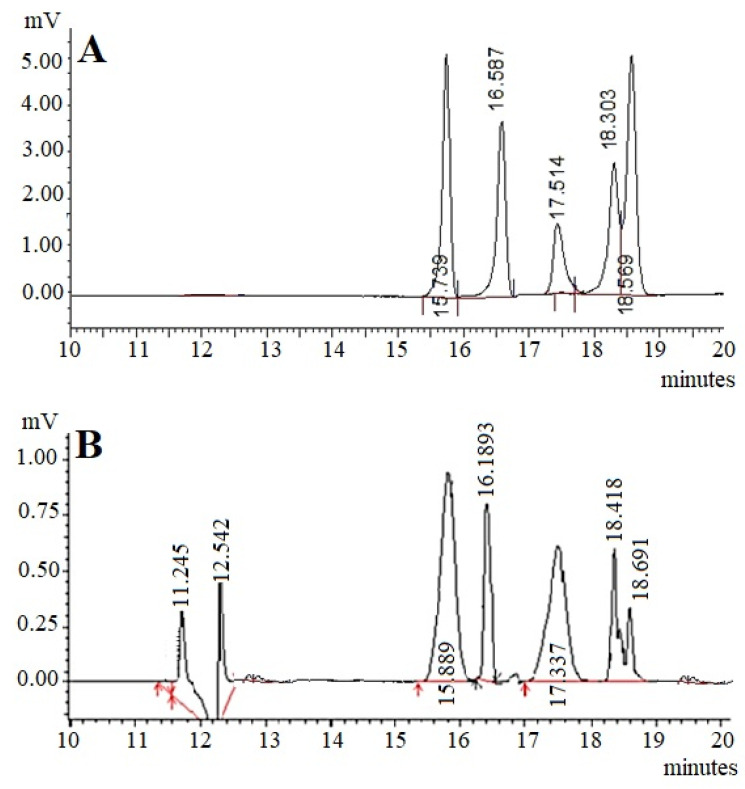
HPLC chromatograms of monosaccharide standard samples (**A**) and hydrolyzed RAP) (**B**). Glucose (RT 15.739 min), galactose (RT 16.587), mannose (RT 17.514), arabinose (RT 18.303), and fucose (RT 18.569). RAP: *Rhododendron arboreum* polysaccharides; RT: Retention time.

The presence of monosaccharides was detected by HPLC analysis according to the retention time of standard monosaccharide samples. The monosaccharide present in the RAP exhibited the same retention time as that of the standard monosaccharides (Figure 1A). The chromatographic analysis revealed that the composition of monosaccharides was found to be glucose (RT 15.739 min), galactose (RT 16.587), mannose (RT 17.514), arabinose (RT 18.303), and fucose (RT 18.569) (Figure 1B).

For statistical analysis of BBD, a total number of 15 runs of experimental conditions including three center points were selected for different combinations. The suitability of model to predict the optimum response value for *R. arboreum* extraction has been evaluated using the optimal conditions chosen. The effects of the independent tested variables (extraction temperature, extraction time, and solid–liquid ratio) on the yield of polysaccharide extraction, including the experimental and predicted values, are shown in Table 1. The model’s appropriateness has been tested and used on the experimental data to explain whether the approaching model will produce bad or misleading results. The different statistical tests were performed in the present study viz. lack of fit tests, the sequential model (sum of squares), and model summary to check the model adequacy to display the maximum yield of polysaccharide. The results for these analyzed tested parameters and the maximum predicted and experimental yields of polysaccharides are in Table 2. The regression analysis was carried out to fit mathematical models to the experimental data, aiming at an optimal region for the responses studied. The most impelling variables on the extraction of RA polysaccharide, viz. extraction temperature, extraction time, and liquid–solid ratio, were investigated. The significance of each coefficient is listed in Table 3. Design Expert Software V 8.0.7.1 was used to analyze and collect data from the experimental runs.

An experimental relationship stated by a second-order polynomial equation with interaction terms was fitted between the obtained results using the BBD model and the input variables. The final (Equation (1)) obtained in terms of coded factors is given below:

Yield of Polysaccharide (%) = 11.64 − 0.375 × A − 0.61 × B 0.695 × C − 1.465 × A × B − 1.255 × A × C − 0.915 × B × C − 1.834 × A^2^ − 1.694 × B^2^ − 2.099 × C^2^. (1)

The results were evaluated using an analysis of variance (ANOVA), and the significance of the experimental results to various models by their corresponding p-values is presented in Table 3. Given the p-values of each model terms, it could be determined that three linear coefficients (A, B, and C), three quadratic coefficients (A^2^, B^2^, and C^2^), and two interactive coefficients (AC and BC) were significant and indicate the pattern of the interactions between the tested variables. The model F-value of 178.56 indicated that the model was highly significant at *p* < 0.0001. The lack of fit F value of 1.87 was insignificant due to the relative pure error (*p* < 0.0702). All together, the exploration and optimization of the fitted response surface may produce poor or misleading results unless the model is fit enough to make it essential to check the suitability of the model [19]. The significance of each coefficient and the strength of interaction between variables are checked by the P-value, and the effects below 0.05 are significant. The higher the value of significance, the better the degree of correlation between the values observed and predicted [20].

**Figure 2:**
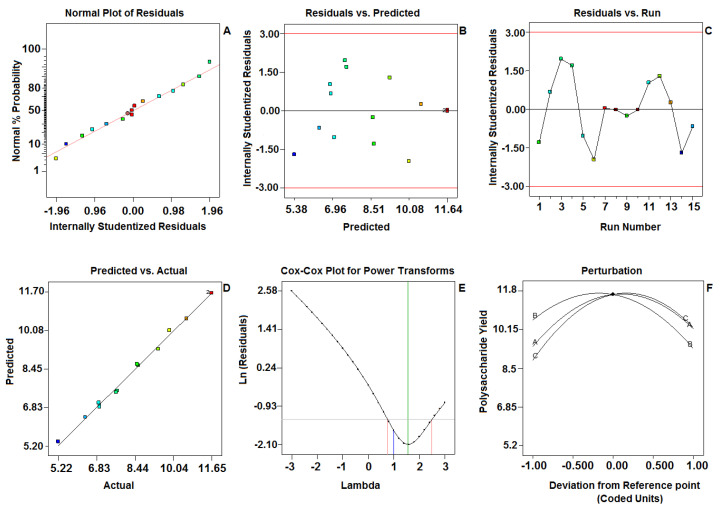
Diagnostic plots for the Box–Behnken model adequacy of the tested variable (predicted vs. actual) against the polysaccharide yield. (**A**) Normal plot of residuals; (**B**) Residuals vs. Predicted; (**C**) Residuals vs. Run; (**D**) Predicted vs. Actual; (**E**) Cox-Cox plot for power transforms; (**F**) Perturbation.

In the present study, the “Pred R-Squared” of 0.9504 is in reasonable agreement with the “Adj R-Squared” of 0.998. Besides this, a very high value of the correlation coefficient (R^2^ = 0.999) exhibited an excellent correlation between the experimental and predicted response values. The low CV value clearly indicated that the variations between the experimental and the predicted values were low and not only showed a high degree of precision, but also had a high degree of reliability in the experiments conducted. Adequate precision measures the ratio of signal to noise. A ratio greater than four is desirable. In the present study, the adequate precision ratio was found to be 39.56, which indicates an adequate signal and confirms that the present model can be used to navigate the design space. The accuracy of the models was measured by checking the diagnostic plots of the experiment and model results. The data are presented in (Figure 2). Figure 2A shows the normal % probability of residual plot for response and was normally distributed, as the points lie rationally close to the straight line and variance deviation was not observed. The present model effectively enhanced the relationship between the process variables and the response. As shown in Figure 2B, the predicted values were very close to the experimental values. The internally studentized residuals versus the experimental runs was analyzed by constructing a satisfying fit of the model, and it indicates that all the data points are placed within the limits (Figure 2C). The values of the predicted and actual response of each run were normally distributed and were near to a straight line (Figure 2D). Box–Cox plots for the power transforms of variables (Figure 2E,F) shows the perturbation of variables in the determined range.

**Figure 3:**
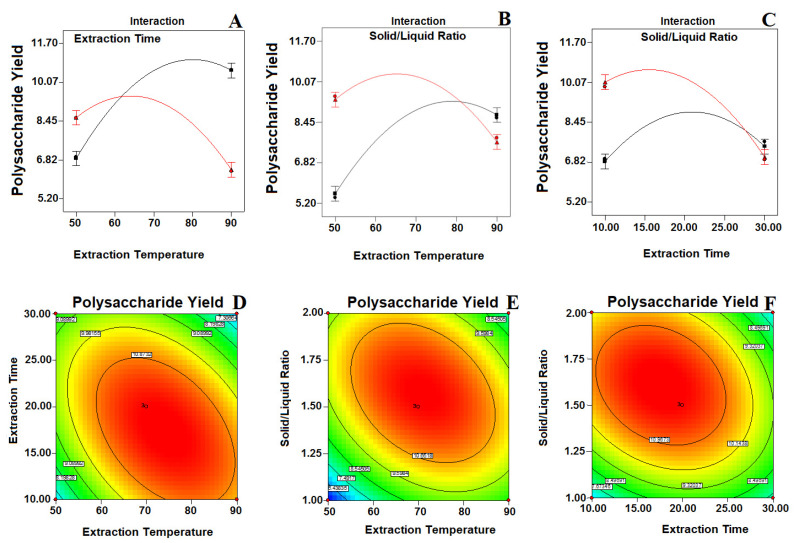
Response surface interaction and contour plots (**A**,**D**) showing the effect of the extraction time and extraction temperature; (**B**,**E**) solid–liquid ratio and extraction temperature; (**C**,**F**) solid liquid ratio and extraction time of the polysaccharide yield. The color intensity (red) in each figure represents the polysaccharide concentration.

The BBD model resulted in three response surface graphs for the extraction of polysaccharides, and these were studied. The interactions between the respective variables are negligible, as shown in the contour plots, although the interactions between the corresponding variables are full size, as shown in the contour plot [21]. From the Figure 3A,B,D,E, it can be seen that the temperature required for extraction confirmed a tremendous linear effect on the polysaccharide yield. The temperature influences the threshold of cavitation, which is responsible for auditory cavitation and further effects on the cavitational nucleus improvement [14,22]. The density and viscosity of the extracts diminish when increasing the temperature from 67 to 90 °C; hence, this facilitates the penetration of the extracting solvent deeper into the matrix of the sample [11]. Ultrasound initially allows the disruption of the cell wall, which increases the solubility and release of polysaccharides into the outside solvent. Likewise, the longer extraction time with ultrasound use could induce polysaccharide degradation and decrease the yield due to the frequent asymmetric collapse of microbubbles [23]. A higher concentration of the solvent–liquid ratio increases the extraction productivity by creating a difference in concentration between the inside of the plant cells and the outside solvent, which in turn increases the rate of mass transfer, resulting in an increased extraction productivity. An increase in the solid–liquid ratio from 1 to 1:66 (g/mL) enhances the yield of polysaccharides (Figure 3B,C,E,F). The above results of the investigation are further supported by the findings of Ahmad and co-workers [14]. Physical effects such as fluid movement and cavitation commotion enhance the material interaction between the desired molecules and the solvent by increasing the solvent’s penetration into the sample matrix, thereby triggering any substantial solvent dissolution and thus increasing the extraction capacity [24].

The measurement of the validity of the model and the maximum yield of polysaccharide was performed using Derringer’s Desirability Point Prediction Tool. The optimal values were found to be 66.75 °C for the temperature of the extract, 19.73 min for the extraction time, and 1.66 mg/L for the solid–liquid ratio, which resulted in a maximum polysaccharide yield of (11.62%), with a desirability value of 1.00. The experiment was conducted in triplicate for the confirmation of the above-mentioned optimum conditions. Such optimized values of the tested parameters were checked under similar conditions (*n* = 6); an average of 11.09% ± 1.65% for the polysaccharide yield was obtained under optimized extraction conditions with a validity of 95.43%. The analytical results showed that the response model was adequate to reflect the optimization expected, and that the model was satisfactory and accurate. The findings are also closely linked to the data obtained from the optimization study using the desirability functions, suggesting that BBD could be used effectively to optimize the polysaccharide extraction parameters. In summary, the extraction methods do play a major role in the isolation of polysaccharides. This will further help in selecting and optimizing the suitable method of extraction for the herbal compounds.

### Evaluation of Antioxidant Activity

The DPPH radical scavenging activity of various RAP extracts are shown in Table 4. RAP exhibited an inhibitory activity against DPPH free radicals in a dose-dependent manner. Free radicals such as lipid peroxides or hydroperoxide radicals play a major role in the propagation of the auto-oxidation of membrane lipids, resulting in lipid peroxidation and leading to cellular toxicity. It has been widely accepted that plant extracts with potential DPPH free radical scavenging activity could serve as potent anti-oxidant agents. Plant free radical scavenging or electron donation ability is mainly attributed to phenolic compounds [25]. This justifies the radical scavenging power of DPPH, as noted in the tested extracts. The results are corroborated with the earlier report, which showed that the DPPH scavenging properties of plant extracts increase with the extract concentration [26].

In Table 4, RAP was found to be effective in scavenging the (2,2′-azino-bis (3-ethylbenzothiazoline-6-sulfonic acid)) ABTS radical. The percentage inhibition of this radical was found to be concentration-dependent. It had an IC_50_ value of 9.62 mg/mL. The ABTS radical-scavenging measuring process, commonly used to measure antioxidant activity, exploits the fact that free ABTS radicals become stable by accepting an antioxidant hydrogen ion and losing their blue color [27,28]. In addition, they have the ability to remove hydroxyl radicals or superoxide in the ABTS assay as well as in the DPPH assay, when antioxidant activity occurs [26,29]. Table 4 demonstrates the NO radical scavenging properties of the polysaccharides. These findings indicated that RAP displayed a higher scavenging potential (*p* < 0.05) at a 25 μg/mL concentration. With the concentration increase, the inhibitory potential of the extracts tested increases. Ascorbic acid, which was used as the regulator, displayed the maximum inhibitory potential overall. The mechanism involved may be due to the presence of phenolic compounds present in the extract, as reported previously [26,30]. In addition, the antioxidant potential of polyphenols is related to the chemical structure, hydroxyl group substitutions, presence of phenolic hydrogen, and the likelihood that the resulting HO and NO radicals will be stabilized by hydrogen donation or electron delocalization [31,32].

## 3. Materials and Methods 

### 3.1. Sample Preparation

The *R arboreum* spp. Arboreum leaves were collected from the Western Himalayan Regions of India and were authenticated by a taxonomist. The leaves were washed with double distilled water, dried at 60 °C, and ground to a fine powder, then sieved through 40-size mesh. The powder was stored in dark polybags and kept in a desiccated environment to prevent any contact of moisture before the experiments. All the other chemicals used for extraction were analytical reagent grade.

### 3.2. Determination and Extraction RAP

The extraction of polysaccharides from *R. arboreum* was carried out by ultrasonic-assisted extraction (UAE), according to the previously described methods [14,33], using an ultrasonic device (Raypa^®^ Instrumentation (Barcelona), Espana) working at a frequency of 60 Hz, with an input power of 220 V and heating power of 550 W, equipped with a time and temperature controller. The percentage yield of polysaccharides (YP %) was calculated by the following (Equation (2)):

YP (%) = weight of crude extract (g)/weight of *R. arboreum* powder (g) × 100 (2)

### 3.3. Monosaccharide Analysis

The identification of monosaccharides was carried out using high performance liquid chromatography (HPLC). The polysaccharide hydrolysis was carried out using the method of Zhang and co-workers [34]. The derivatives of monosaccharides were prepared according to the method of Kan and colleagues [35]. The analysis was carried out on a C_18_ column (4.6 mm × 250 mm, 5 µm) by HPLC (Shimadzu, Japan). The composition of the mobile phase consists of acetonitrile and 0.05 M phosphate buffer (pH = 6.8) (25:75, *v/v*) at a 1 mL flow rate. The injection volume of 10 µL and the detection was carried out at a wavelength of 245 nm.

### 3.4. Process Optimization

A response surface methodology (RSM) was used to optimize and evaluate the maximum percent yield of polysaccharide from RA. The effects of the three independent variables (extraction temperature of 50–90 °C, extraction time of 10–30 min, and solid–liquid ratio of 1–2 g/mL) on the response (PY) were investigated, and the optimal conditions were check using the Box–Behnken experimental design of RSM to improve the yield of the polysaccharide percentage from RA. Three independent process variables, including extraction temperature (A), extraction time (B), and solid–liquid ratio (C), significantly affected the extraction productivity, and the optimal range of each tested variable was determined. Three repeats of the central run, leading to 15 sets of experiments, enabled each experimental response to be optimized. The experimental design also consists of three center points in 15 experiments in order to allow the estimation of pure error, which allows calculating the response of intermediate levels and enables the estimation of the system performance at any experimental point within the studied range.

Using the program Design Expert V 8.0.7.1, a Box–Behnken design was implemented with three factorial points and three independent variables to statistically optimize the extraction process. The experimental designs of the code and the actual levels of each factor are shown in Table 1. The significance in the model was evaluated by an analysis of variance (ANOVA). Graphical and numerical analyses were used to optimize the processing conditions based on the model desirability features. The experiment was finally replicated under optimum values according to the Derringer’s desirable response surface methodology prediction tool for the extraction temperature, time, and solid–liquid ratio, which should result in the maximum polysaccharide yield percentage.

### 3.5. Statistical Analysis

An analysis of variance (ANOVA) and a multiple regression analysis were conducted for fitting the model using the Box–Behnken design. The experimental analyses and calculations were carried out in the Graph Pad Prism V6, La Jolla, CA. USA). After fitting the data to the models, the data produced were used for plotting the response surfaces, interference, and contour plots. The differences between the means were considered statistically significant at *p* < 0.05.

### 3.6. Anti-Oxidant Activity

#### 3.6.1. Scavenging Effect on 2, 2-Diphenyl-1-picryl Hydrazyl Radical (DPPH)

The free radical scavenging ability of the RAP extracts were evaluated by the DPPH method, a previously reported method [36]. Ascorbic acid was used as a standard and was dissolved in distilled water to achieve the concentration of 1 mg/mL, followed by serial dilutions (10–50 μg/mL) (Equation (3)).

% Inhibition = (Ac − As)/Ac × 100(3)

where Ac and As represent the absorbance of the control and the samples, respectively.

#### 3.6.2. ABTS + Radical Scavenging Effect

The assay was based on a previously reported method [37]. Ascorbic acid was used as a standard and the absorbance was measured at 734 nm. The decrease in absorption was used to calculate the values for the scavenging effect. The above equation served to evaluate the percentage radical scavenging activity of each sample.

#### 3.6.3. Nitric Oxide (NO) Scavenging Activity

The scavenging activity of NO was determined according to the previous method [26,38]. The pink chromophore that was produced with sulphanilamide during the diazotization of nitrite ions and the consequent coupling with α-naphthyl ethylene diamine was recorded at 540 nm spectrophotometrically. Ascorbic acid has been used as a reference standard. The NO scavenging capacity (%) was calculated using the formula above.

## 4. Conclusions

In the present study, the response surface methodology was followed, optimizing the extraction conditions for the maximum polysaccharide yield, and showed that a significant enhancement of the polysaccharide yield can be achieved with the use of UAE. Overall, the BBD model of RSM was able to predict the RAP yield and provide valuable tools for evaluating the different process configurations. To optimize the individual and interactive effects of the tested variables, such as extraction temperature, time, and solid–liquid ratio, on the RAP, the BBD has been successfully implemented using UAE and the assessment of its antioxidant effect. The model statistics showed that, with the experimental data, the model obtained was reliable, adequate, and precise. ANOVA’s higher coefficient of determination (R^2^ = 0.999) showed a reasonable adequacy of the second-order polynomial regression model developed. The Desirability Prediction Tool of Derringer was achieved under the optimal extraction conditions (extraction temperature 66.75 °C, extraction time 19.72 min, and liquid–solid ratio 1.66 mL/g) with a desirability value of 1, yielding the highest percentage of polysaccharide (11.56%). The expected yield of polysaccharide was closely related to the experimental values under same optimized conditions. The RAP has also shown that *R. arboreum* has a marked anti-oxidant activity.

## 5. Limitations and Future Prospects

There has been much attention given to developing drugs from polysaccharides in recent years. Further studies are warranted to scale up the method of extraction for the commercial usage of the polysaccharides. The study’s potential opportunities include chemical characterization, purification, and functional research. The development of anti-cancer and antioxidant drugs from polysaccharides has received incredible attention over the last decade. Several in vitro experiments appear to be beneficial, yet to confirm such findings, in vivo tests need to be performed. Further studies are necessary to explore the clinical implications of such findings. More efficient and cost-effective methods for modifying and preparing polysaccharides remain major challenges, and thus an important amount of research is yet to come.

## Figures and Tables

**Table 1:** The Box–Behnken response surface design and corresponding response values.

Run	Temp. (°C)	Time (min)	Solid/Liquid (g/mL) Ratio	RAP (%)		Residual
				Actual	Predicted	
**1**	50	10	1.5	6.95	6.885	0.065
**2**	90	10	1.5	10.59	10.56	0.025
**3**	50	30	1.5	8.57	8.59	−0.025
**4**	90	30	1.5	6.35	6.41	−0.065
**5**	50	20	1	5.22	5.38	−0.165
**6**	90	20	1	8.52	8.64	−0.125
**7**	50	20	2	9.41	9.28	0.125
**8**	90	20	2	7.69	7.52	0.165
**9**	70	10	1	6.95	6.85	0.1
**10**	70	30	1	7.65	7.46	0.19
**11**	70	10	2	9.88	10.07	−0.19
**12**	70	30	2	6.92	7.02	−0.1
**13**	70	20	1.5	11.65	11.64	0.0066
**14**	70	20	1.5	11.64	11.64	−0.0033
**15**	70	20	1.5	11.64	11.64	−0.0033

**Table 2:** Sequential model fitting for the yield of polysaccharide.

Model (Sequential) Sum of Squares					
Source	Sum of Squares	Df	Mean Square	F Value	Prob > F
Mean vs. Total	1120.26	1	1120.26		
Linear vs. Mean	7.96	3	2.65	0.56	0.6549
2FI vs. Linear	18.24	3	6.08	1.42	0.3074
Quadratic vs. 2FI	34.19	3	11.37	303.006	<0.0001
Cubic vs. Quadratic	0.18	3	0.06	1876	0.0005
Residual	6.67	2	3.33		
Total	1180.77	15	78.72		
**Lack of Fit Tests**					
Linear	52.53997	9	5.837775	175,133.2	<0.0001
2FI	34.30607	6	5.717679	171,530.4	<0.0001
Quadratic	0.1876	3	0.062533	1876	0.0005
Cubic	0	0			
Pure Error	6.67 × 10^−5^	2	3.33 × 10^−5^		
**Model Summary Statistics**					
**Source**	**SD**	***R^2^***	**Adjusted *R^2^***	**Predicted *R^2^***	**Press**
Linear	2.18	0.13	−0.10	−0.41	85.66
2FI	2.07	0.43	0.001	−0.15	69.80
Quadratic	0.19	0.999	0.99	0.95	3.01
Cubic	0.005	0.999	0.99		

**Table 3:** Analysis of the variance and regression coefficients of the calculated surface quadratic model polysaccharide yield.

Source	Sum of Squares	Coefficient Estimate	Df	Mean Square	SEM	F Value	*p*-Value
Model	60.31	11.64	9	6.70	0.11	178.56	<0.0001
A-Extraction Temperature	1.12	0.37	1	1.12	0.068	29.97	0.0028
B-Extraction Time	2.97	−0.61	1	2.97	0.068	79.31	0.0003
C-Solid/Liquid Ratio	3.86	0.69	1	3.86	0.068	102.95	0.0002
AB	8.58	−1.46	1	8.58	0.097	228.72	<0.0001
AC	6.30	−1.25	1	6.30	0.097	167.85	<0.0001
BC	3.34	−0.91	1	3.34	0.097	89.22	0.0002
A^2^	12.42	−1.83	1	12.42	0.10	330.94	<0.0001
B^2^	10.59	−1.69	1	10.59	0.10	282.35	<0.0001
C^2^	16.27	−2.09	1	16.27	0.10	433.48	<0.0001
Residual	0.18		5	0.037			
Lack of Fit	1.876		3	0.062			0.0702
Cor Total	60.50		14				

**Table 4:** Antioxidant activity of *Rhododendron arboreum* polysaccharides (RAP) on 2,2-diphenyl-1-picryl hydrazyl radical (DPPH), 2,2′-azino-bis (3-ethylbenzothiazoline-6-sulfonic acid) (ABTS), and Nitric oxide (NO).

DPPH						
Ascorbic Acid				RAP Extract		
S. No.	Conc. (µg/mL)	% Inhibition (Mean± SEM)	IC_50_ (µg/mL)	Conc. (µg/mL)	% Inhibition (Mean ± SEM)	IC_50_ (µg/mL)
**1**	5	41.21 ± 1.34	8.84	5	31.26 ± 1.51	27.66
**2**	10	52.59 ± 1.59		10	42.56 ± 1.29	
**3**	15	64.12 ± 1.21		15	53.22 ± 1.45	
**4**	20	73.11 ± 1.15		20	63.44 ± 1.56	
**5**	25	83.13 ± 1.77		25	71.29 ± 1.88	
**ABTS**						
**1**	5	38.22 ± 1.56	9.62	5	26.22 ± 1.09	31.26
**2**	10	51.60 ± 1.44		10	36.85 ± 1.22	
**3**	15	63.12 ± 1.13		15	49.42 ± 1.51	
**4**	20	76.12 ± 1.22		20	59.11 ± 1.74	
**5**	25	90.31 ± 2.09		25	71.88± 1.91	
**NO**						
**1**	5	36.29 ± 1.09	10.32	5	18.65 ± 1.31	38.24
**2**	10	49.22 ± 1.38		10	29.55 ± 1.22	
**3**	15	61.85 ± 1.71		15	41.22 ± 1.19	
**4**	20	74.98 ± 1.88		20	56.66 ± 1.48	
**5**	25	88.65 ± 1.99		25	62.35 ± 1.63	
